# Abnormal Pulmonary Function and Respiratory Muscle Strength Findings in Chinese Patients with Parkinson’s Disease and Multiple System Atrophy–Comparison with Normal Elderly

**DOI:** 10.1371/journal.pone.0116123

**Published:** 2014-12-29

**Authors:** Yao Wang, Wei-bo Shao, Li Gao, Jie Lu, Hao Gu, Li-hua Sun, Yan Tan, Ying-dong Zhang

**Affiliations:** 1 Department of rehabilitation medicine, Nanjing Brain Hospital, Nanjing medical university, Nanjing, China; 2 Department of neurology, Renji Hospital, Shanghai Jiaotong University School of medicine, Shanghai, China; 3 Department of neurology, Nanjing Brain Hospital, Nanjing medical university, Nanjing, China; 4 Department of respiratory, Nanjing First Hospital, Nanjing medical university, Nanjing, China; 5 Department of neurology, Nanjing First Hospital, Nanjing medical university, Nanjing, China; Hospital General Dr. Manuel Gea González, Mexico

## Abstract

**Background:**

There have been limited comparative data regarding the investigations on pulmonary and respiratory muscle function in the patients with different parkinsonism disorders such as Parkinson’s disease (PD) and multiple system atrophy (MSA) versus normal elderly. The present study is aiming to characterize the performance of pulmonary function and respiratory muscle strength in PD and MSA, and to investigate the association with severity of motor symptoms and disease duration.

**Methods:**

Pulmonary function and respiratory muscle strength tests were performed in 30 patients with PD, 27 with MSA as well as in 20 age-, sex-, height-, weight-matched normal elderly controls. All the patients underwent United Parkinson’s disease rating scale (UPDRS) or united multiple system atrophy rating scale (UMSARS) separately as diagnosed.

**Results:**

Vital capacity, forced expiratory volume in 1 second and forced vital capacity decreased, residual volume and ratio of residual volume to total lung capacity increased in both PD and MSA groups compared to controls (*p*<0.05). Diffusing capacity was decreased in the MSA group, compared with PD and normal elderly control groups (*p*<0.05). Respiratory muscle strength was lower in both PD and MSA groups than in controls (*p*<0.05). The values representing spirometry function and respiratory muscle strength were found to have a negative linear correlation with mean score of UPDRS-III in PD and mean score of UMSARS-I in MSA. Respiratory muscle strength showed a negative linear correlation with the mean score of UMSARS-II and disease duration in MSA patients.

**Conclusions:**

These findings suggest that respiratory dysfunction is involved in PD and MSA. Respiratory muscle strength is remarkably reduced, and some of the parameters correlate with disease duration and illness severity. The compromised respiratory function in neurodegenerative disorders should be the focus of further researches.

## Introduction

Parkinson’s disease (PD) and multiple system atrophy (MSA) are the most common neurodegenerative disorders perceived as diseases of the motor system. However, there also are a number of non-motor symptoms in these disorders that have recently received a lot of interest [1]–[2], including autonomic, sensory, neuropsychiatric and cognitive dysfunction for PD [1], [3] and autonomic failure, sleep disturbance and olfactory abnormalities for MSA [2].

Because the clinical presentation of dysautonomia overlaps, results of studies investigating the differential diagnostic value of these two neurodegenerative disorders by laboratory investigations mostly evaluating cardiovascular system [4], urogenital system [5]–[6], sympathetic sudomotor system [7] have been contradictory. But the characteristics of the respiratory system have not yet been widely investigated.

The pneumologic problems are one of the most common causes of death in patients with PD [8], most of whom rarely have complaints despite the final course of disease [9], and the disturbance is usually unnoticed by clinicians.

Several lines of evidence have suggested that pulmonary abnormalities are associated with PD. Pulmonary function tests, respiratory muscle function measurements and diaphragmatic electromyogram have revealed that PD patients suffer obstructive, restrictive or mixed pattern of ventilatory dysfunction, upper airway dysfunction, and diminished strength of respiratory muscles [9]–[10] even at the early stages [11], possibly due to central or peripheral desynchronization of the respiratory muscles [12], or involvement of levodopa medication [13]–[14].

Respiratory symptoms that the patients of MSA suffer include stridor, sleep-related breathing disorders, and respiratory insufficiency [2], with brainstem degeneration playing a probably main role [15]. These symptoms are now included in the list of additional and supportive features of the new consensus diagnostic criteria [16]. Nevertheless, the pattern of pulmonary abnormalities, or the performance of respiratory muscles have not yet been well described in MSA.

Although pulmonary function tests have already been investigated in several studies with PD [9]–[13] and MSA [17], to the best of our knowledge, there are limited comparative data regarding these investigations in patients with these two diseases versus healthy controls. The present study is aiming to characterize the performance of pulmonary and respiratory muscle function in PD and MSA and to investigate the association of these changes with motor symptom severity and disease duration, in order to find an approach to discriminate between these two diseases and to explain the possible mechanism.

## Material and Methods

### Patients

This retrospective analysis included thirty patients with PD and twenty-seven with MSA, recruited consecutively from Nanjing Brain Hospital and Nanjing First Hospital affiliated to Nanjing Medical University between June 2012 and June 2014, Twenty age-, sex-, height- and weight-matched healthy controls in this case-control study were also included.

Differential diagnosis was based on the UK Brain Bank criteria for PD [18] and the new consensus criteria for MSA [16]. Data were collected on age, sex, height, weight and disease duration. Disease severity was evaluated by the neurologist who treated those patients before the respiratory function testing performed using the Hoehn and Yahr (H & Y) scale for PD cases, and the united Parkinson’s disease rating scale (UPDRS) or united multiple system atrophy rating scale (UMSARS) separately as diagnosed. All the subjects with PD and twenty patients diagnosed with MSA received levodopa medication routinely for better compliance. All participants received verbal and written information about the purpose and process of our research which approved by the Ethical Committees of Nanjing First Hospital. And the individual in this manuscript has given written informed consent (as outlined in *PLOS* consent form) to publish these case details.

Exclusion criteria included any clinical respiratory or cardiovascular diseases, history of lung surgery, recent respiratory tract infection, anemia, dementia, autonomic failure caused by diabetes, renal dysfunction, amyloid degeneration and stabilizer abuse. Patients who complained of any breathing discomfort were also excluded.

### Instruments

This study was performed at the respiratory department in Nanjing First Hospital. Each subject took the Respiratory function tests (RFT) in a sitting position using a spirometer (Masterscreen-body/Diff+IOS+APS, Jaeger, German) between 9 am and 3 pm with a stable room temperature of 25–28 centigrade. Lung volumes were measured by body plethysmography. The carbon monoxide diffusion capacity (DLCO) was measured with a gas mixture containing air, 10% helium, and 0.3% carbon monoxide; each measurement was adjusted to the standard temperature and pressure. The best of three technically acceptable tests was included in the study. All the tests were performed by one single physician. All the PD patients took tests in the “on” state.

### Measurements

The parameters we collected from the evaluation of respiratory function including lung volume, spirometry, diffusing capacity and respiratory muscle strength were as follows:

#### Lung volume

Vital capacity (VC), total lung capacity (TLC), residual volume (RV), ratio of residual volume to total lung capacity (RV%TLC);

#### Spirometry

Forced vital capacity (FVC), forced expiratory volume in 1 second (FEV_1_), ratio of FEV_1_ to FVC (FEV_1_%FVC), peak expiratory flow (PEF), maximum voluntary ventilation (MVV), maximal expiratory flow after expiration of 50% of FVC (MEF50);

#### Diffusing capacity

Carbon monoxide diffusion capacity (DLCO);

#### Respiratory muscle strength

Maximum of inspiration pressure (MIP), maximum of expiration pressure (MEP), occlusion pressure (P_0.1_).

All the results were expressed as percentages of predicted values, except for RV%TLC. Predicted values were derived from the recommendations of “the normative values of pulmonary function testing in Chinese adults” [19] by CareFusion JLAB software V5.3x. Lung volume, spirometry, and diffusing capacity were performed according to ATS/ERS criteria [20]–[22], while respiratory muscle strength was determined as previously described by Black and Hyatt [23]. Furthermore, the liner correlation between the values with the scores of two scales and duration of disease were also collected.

### Standardization

Patients with respiratory dysfunction were diagnosed as central obstructive ventilation with FEV_1_<80% and FEV_1_%FVC<80%, peripheric obstructive pattern with MEF50<70%, restrictive ventilation with FVC<80%, and FEV_1_%FVC>80% [9], and diffusion dysfunction with DLCO<80% [22].

### Statistical analysis

All the results were expressed as mean±SD and were examined for the homogeneity of variance. Statistical analysis was performed using SPSS 18.0 software. Comparisons between multiple groups were evaluated by one way analysis of variance (*ANOVA*) followed by the *LSD* procedure. Correlations were assessed by calculating *Pearson* correlation coefficients. A value of *P*<0.05 was considered to be statistically significant.

## Results

### Demographics and group description

In this study, 30 (16 male) with PD, 27 (14 male) with MSA, and 20 (10 male) healthy elderly controls were evaluated. There were 20 of probable and 7 of possible MSA, 18 of MSA-P, 5 of MSA-C and 4 of MSA-P+C for the subtype classification [16]. The demographic details of age, sex, height, weight and disease duration are shown in Table 1, with no significant differences were between each two groups (*P*>0.05).

**Table 1:** Clinical and Demographic Features for PD, MSA and Controls.

Characteristic	PD	MSA	Controls	*P*-value
	N = 30	N = 27	N = 20	
Male, n%	16 (53.3)	14 (51.9)	10 (50.0)	0.963
Age, mean±SD	61.80±4.20	59.85±5.20	60.85±4.87	0.307
Height, cm, mean±SD	163.83±7.73	166.78±7.02	164.70±7.29	0.306
Weight, kg, mean±SD	62.77±7.82	64.30±7.87	63.85±9.50	0.776
Disease duration, years, mean±SD	4.90±3.10	3.63±1.45	0	0.061
Hoehn and Yahr stage, n%		–	0	
2.0	5 (16.7)			
2.5	13 (43.3)			
3.0	10 (33.3)			
4.0	1(3.3)			
5.0	1(3.3)			
Score of UPDRS-III	32.47±12.99	–	0	
Score of UMSARS-I	–	18.33±4.67	0	
Score of UMSARS-II	–	22.93±6.72	0	

PD:Parkinson’s Disease;MSA:Multiple System Atrophy;UPDRS-III:motor section of United Parkinson’s Disease Rating Scale; UMSARS-I:The historical part of United Multiple System Atrophy Rating Scale;UMSARS-II: The motor section of United Multiple System Atrophy Rating Scale.

40% (12/30) of PD and all of the MSA patients showed autonomic failure: orthostatic hypotension exhibited in 13.3% (4/30) of PD and 88.9% (24/27) of MSA, 33.3% (10/30) of PD and 85.19% (23/27) of MSA patients complained about urinary incontinence, while 6.7% (2/30) of PD and 44.4% (12/27) of MSA cases suffered sympathetic sudomotor disturbance.

The H & Y stage was II to V for PD patients. The mean scores of motor section of UPDRS (section III), UMSARS (section II) and historical part of UMSARS (section I) were 32.47±12.99, 18.33±4.67 and 22.93±6.72, respectively.

### Respiratory function tests

Seventy-seven subjects underwent the respiratory function tests including pulmonary function and respiratory muscle strength tests. Three patients with PD and two with MSA did not fulfill the TLC, RV, or the diffusion capacity portions of the tests.

#### Lung volume

**Figure 1:**
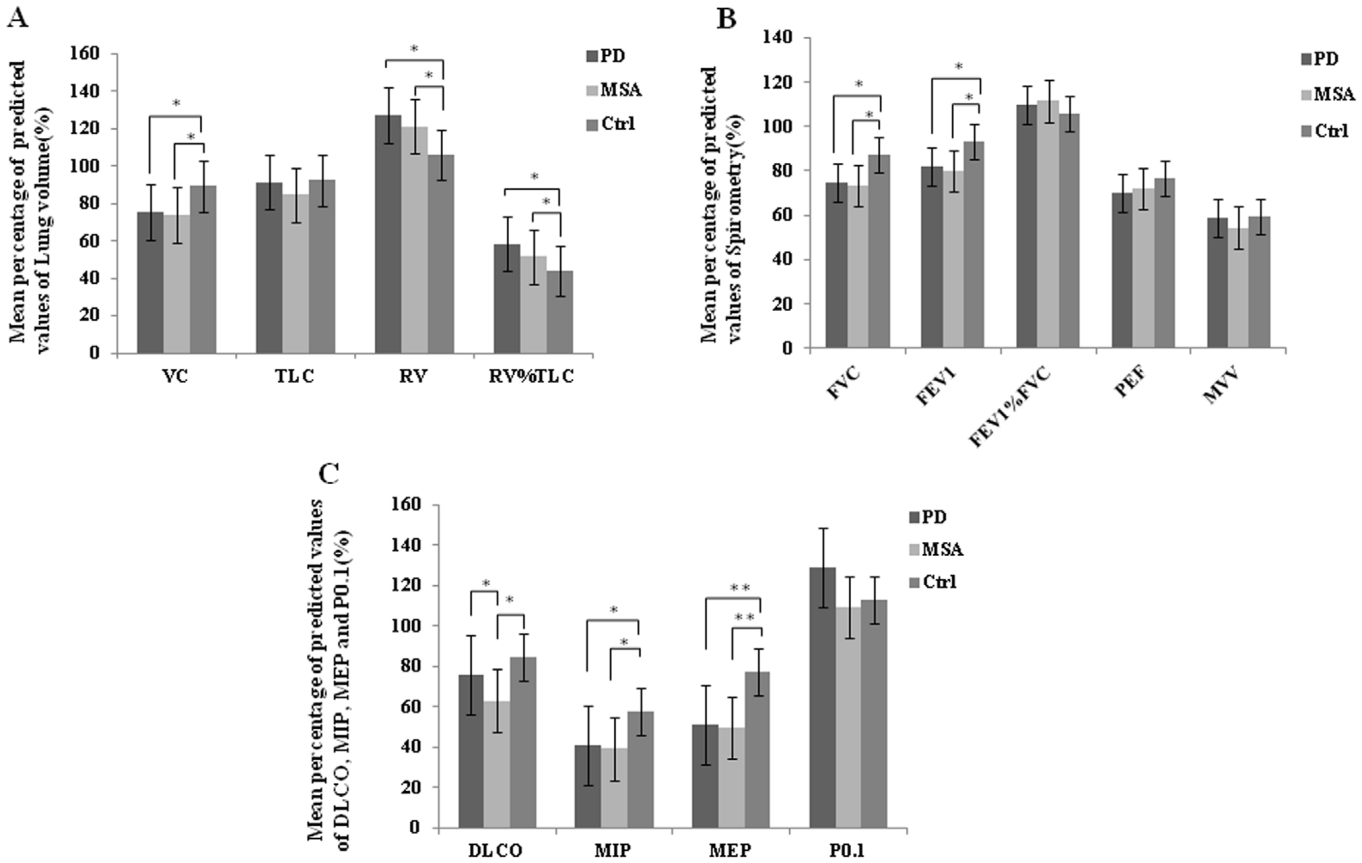
The mean percentage of predicted values of (A) lung volume, (B) spirometry and (C) diffusion capacity and respiratory muscle strength in Parkinson’s disease (PD), multiple system atrophy (MSA) and healthy controls (Ctrl). Asterisks indicate statistically significant differences between any of two groups on one-way ANOVA followed by the LSD procedure. Statistic threshold: *P<0.05, ** = P<0.001.

Fig. 1A showed that the patients groups had lower VC (PD: 74.77±13.92%, MSA: 72.99±14.46%), higher RV (PD: 122.66±26.54%, MSA: 109.21±31.25%) and RV%TLC (PD: 56.67±20.10%, MSA: 51.21±8.74%) than healthy controls (VC: 88.64±8.44%, RV: 104.09±19.41%, RV%TLC: 44.34±5.62%), with significant differences between the groups (*P*<0.05). While there were no significant differences in measurement of TLC between the three groups.

#### Spirometry

The mean percentage of predicted values of FEV_1_ and FEV_1_%FVC seemed normal (>80%) in both patient groups. FEV_1_ (PD: 81.23±15.51%, MSA: 80.60±11.82%) and FVC (PD: 75.25±14.53%, MSA: 75.05±13.06%) appeared reduced (Fig. 1B) with significant differences between the patient groups and controls (FEV_1_: 92.40±13.39%, FVC: 87.57±9.00%) (*P*<0.05). Nevertheless, there were no obvious differences in PEF, MVV and MEF50 whithin the three groups.

#### The type of ventilation dysfunction

**Figure 2:**
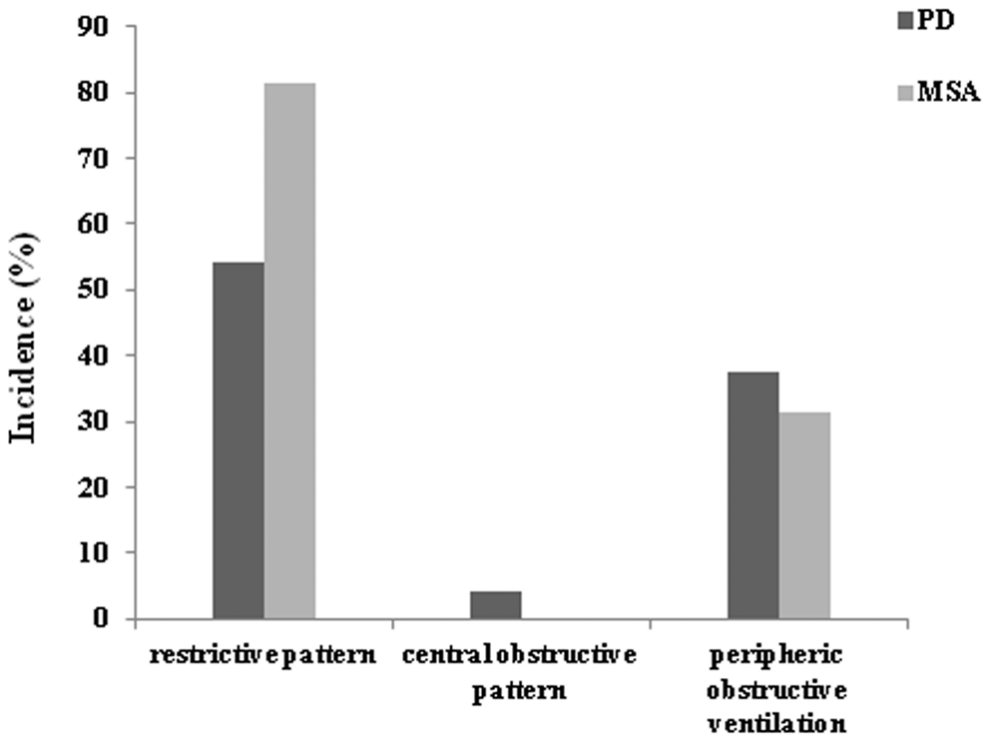
Incidence of restrictive or obstructive pattern of ventilatory dysfunction in Parkinson’s disease (PD) and multiple system atrophy (MSA).

More than two thirds of the patients had spirometry data showing a mild to moderate ventilation dysfunction. A restrictive pattern was found in 56.7% (17/30) of patients with PD and 63.0% (17/27) with MSA, a central obstructive pattern was found in 3.3% (1/30) of PD patients but in none of the MSA patients, and peripheric obstructive ventilation was found in 43.3% (13/30) of PD patients and 29.6% (8/27) of MSA patients (Fig. 2).

#### Diffusion capacity and Respiratory muscle strength

Fig. 1C showed that DLCO reduced in MSA (65.28±12.96%) compared to PD (76.69±15.08%, *P*<0.05) and controls (83.02±6.50%, *P*<0.001).

The maximum pressure of inspiration and expiration decreased markedly in both PD and MSA (MIP: 42.78±22.31% for PD and 45.83±19.93% for MSA; MEP: 52.34±22.13% for PD and 55.57±20.31% for MSA), as compared to controls [MIP: 58.61±18.58% (*P*<0.05), and MEP: 76.16±19.84% (*P*<0.001)]. However, there were no significant differences either between the patients groups for MIP and MEP, or among the subjects for the mean value of P_0.1_ (Fig. 1C).

### Correlation

#### The correlation between RFT values and motor section of UPDRS (part III)

VC, FVC, FEV_1_, MEP, PEF and MIP in PD patients were negatively correlated with their UPDRS-III scores [r = −0.495, –0.473, –0.407, –0.455 (all P<0.05), –0.540, –0.558 (both P<0.01), see Table 2].

**Table 2:** The Liner Correlation between Main Part of Respiratory Function Tests (RFT) Values with Severity of Motor Symptoms and Disease Duration of Patients with Parkinson’s Disease (PD) and Multiple System Atrophy (MSA).

	Lung volume			Spirometry			Diffusion capacity	Respiratory muscle strength	
	VC	FVC	FEV_1_	PEF	MVV	MEF50	DLCO	MIP	MEP
**UPDRS-III**	−0.495*	−0.473*	−0.407*	−0.540**	−0.305	−0.242	−0.010	−0.558**	−0.455*
**DD of PD**	0.221	0.144	0.235	0.234	−0.085	0.231	0.594**	0.140	−0.113
**UMSARS-I**	−0.590*	−0.536*	−0.501*	−0.473*	−0.338	−0.326	−0.265	−0.500**	−0.586**
**UMSARS-II**	−0.331	−0.367	−0.182	−0.329	−0.357	−0.025	−0.129	−0.416*	−0.546**
**DD of MSA**	−0.378	−0.352	−0.223	−0.184	−0.113	−0.187	−0.065	−0.404*	−0.531**
**MIP of PD**	0.546**	0.613**	0.482*	0.423*	0.417*	0.223	0.037	1	0.511*

VC, vital capacity; FVC, forced vital capacity; FEV_1_, forced expiratory volume in 1 second; PEF, peak expiratory flow; MVV, maximum voluntary ventilation; MEF75, maximal expiratory flow after expiration of 75% of FVC; MIP, maximum of inspiration pressure; MEP, maximum of expiration pressure; UPDRS-III: motor section of united Parkinson’s disease rating scale; DD: disease duration; UMSARS-I: historical part of united multiple system atrophy rating scale; UMSARS-II: motor section of united multiple system atrophy rating scale; PD: Parkinson’s disease; MSA: multiple system atrophy. * *P*<0.05, ** *P*<0.01.

#### The correlation between RFT values and historical & motor section of UMSARS

In the MSA group, linear negative correlations between VC, FVC, FEV_1_, PEF, MIP and MEP and UMSARS-I scores were observed [r = −0.590, –0.536, –0.501, –0.473 (respectively, all P<0.05), –0.500, –0.586 (P<0.01), see Table 2], while MIP and MEP were negatively correlated UMSARS-II scores [r = −0.416 (P<0.05), –0.546 (P<0.01), respectively, see Table 2]. However, no linear correlation was detected between DLCO and UMSARS-I or II scores (For I: r = −0.265, P>0.05; For II: r = −0.129, P>0.05, see Table 2).

#### The correlation between RFT values and disease duration in PD and MSA

Only the predicted value of MIP and MEP showed an obvious negative correlation with disease duration in MSA [r = −0.404 (P<0.05), –0.531 (P<0.01), respectively, see Table 2], while there was a close positive linear correlation between DLCO and disease duration in PD (r = 0.594, P<0.01, see Table 2).

## Discussion

This study used tests of pulmonary function and respiratory muscle strength to demonstrate that Chinese patients with PD and MSA suffered respiratory disturbance. Pulmonary function tests in PD group showed abnormal levels in the mean values of lung volume and ventilation function than healthy elderly controls (Fig. 1A). Based on the standardization of ventilation disturbance [24], PD patients were found to have restrictive pattern at average level. While high incidence of restrictive and peripheric obstructive pulmonary functional status was found in PD cases (Fig. 2), it has also been revealed that the more seriously the patients suffered motor symptoms, not the longer course of disease, the worse they performed on the tests in the present study (Table 2).

Similar to the PD group, the average values of lung volume and ventilation function in MSA patients were different statistically from control group. Most patients with MSA suffered a restrictive and/or peripheric obstructive ventilation disturbance (Fig. 2). Interestingly, DLCO, which reflected the function of gas exchange, was significantly reduced in the MSA group compared to the rest groups (Fig. 1B), with no linear correlation with disease duration or severity of symptoms (Table 2).

Respiratory muscle strength tests revealed a remarkable decrease of MIP and MEP in both PD and MSA groups (Fig. 1C). Furthermore, the predicted values of MIP and MEP showed a negative linear correlation with severity of motor symptoms of PD and MSA. MIP and MEP also presented a close negative relation with course of disease in MSA (Table 2).

Impairment of FVC, FEV_1_, MIP and MEP were demonstrated in PD patients in this study, as shown in some publications [11], [25]–[26], but not consistent with the findings of others [9]. Furthermore, respiratory disturbance had been indicated without anti-parkinsonism pharmacological withdrawal in our patients because studies reported conflicting results and views on the role of levodopa in the treatment of respiratory disturbances in PD [11], [13]–[14], [26]–[27].

Nevertheless, there have been limited data regarding the pattern of pulmonary abnormalities or the performance of respiratory muscles in MSA patients. Shimohata T and colleagues [17] performed conventional spirometry and found that most MSA subjects had normal VC and FEV_1_ values, without correlation coefficients between disease duration and variables, which differed from our results.

There are several possible mechanisms for the respiratory dysfunction in PD, which have already been discussed in previous studies. An involvement of the upper airway muscles which changes the airflow resistance leading to the flow oscillation causes an upper airway dysfunction in extrapyramidal diseases [25]. Apart from the involvement of upper airway muscles, tremor or jerky movements of the diaphragm may also be associated with pulmonary impairment in PD [10]. Furthermore, reduction of expiratory muscle strength possibly contributes to the pulmonary obstructive defects [12]. In our present study we found linear positive correlations between MIP and VC, FVC, FEV_1_, PEF, MVV and MEF75 (Table 2) in PD patients, whose major ventilation dysfunction was restrictive. Therefore, we suggest that the abnormal ventilation tests in PD patients might be attributed to the reduced inspiratory muscle strength.

The role of levodopa’s effect on respiratory function had been debated for years. Some studies described it as having an adverse dopaminergic effect [14], whereas others described it as a wearing-off of PD symptoms during the “off” period [13]. However, a recent paper reported that all pulmonary function test parameters significantly improved after levodopa [11]. A meta-analysis suggested that levodopa significantly improved FVC and PEF, providing indirect evidence regarding the efficacy of levodopa on restrictive parameters [28]. However, we propose that levodopa does play an important role in generation of respiratory dysfunction in PD. Further studies containing patients without wearing-off symptoms are needed.

Notably, MSA patients demonstrated a lower diffusion capacity than PD patients and controls, which indicates a different mechanism. The capacity of the lung to exchange gas across the alveolar-capillary interface is determined mainly by the thickness and area of the alveolar capillary membrane, the concentration and binding properties of hemoglobin (Hb) in the alveolar capillaries and the volume of blood in capillaries supplying ventilated alveoli [22]. We carefully excluded any history of clinical respiratory or cardiovascular disease, lung surgery, recent respiratory tract infection or anemia, thus the volume of blood in capillaries supplying ventilated alveoli is more likely to contribute to the poor cardiovascular reflex performance in MSA. Furthermore, decreased heart rate variability and severe hypotensive responses were recently observed in MSA patients regardless of age and disease duration [4], which supported our findings that the value of DLCO has no close correlation with disease duration or severity of motor symptoms.

A reduction in respiratory muscle strength is demonstrated in both PD and MSA groups without significant differences between them (Fig. 1C), which indicates that the two disorders may share the same mechanism. As far as we know, automatic breathing depends on a central pattern generator that is located in the lower brainstem. Neurons located in the dorsolateral pons and the ventrolateral medulla, which are mostly chemosensitive glutamatergic neurons, have been implicated in control of cranial and spinal motoneurons that drive the muscles of the upper respiratory airway and the respiratory pump [15]. Chemosensitive glutamatergic and serotonergic neurons located just beneath the ventral medullary surface [29], corresponding to the human arcuate nucleus (ArcN) and participating in local network interactions involved in respiratory chemosensitivity [30], are depleted in MSA patients [31]. In addition, changes in the medulla where the earliest pathological variation of PD happens based on the Braak staging hypothesis, implies early pre-motor dysfunctions [32]. Therefore, it is possible that there is depletion of the chemosensitive glutamatergic neurons in the dorsolateral pons and the ventrolateral medulla in both PD and MSA patients which may trigger the impairment of respiratory muscle control. Further pathological studies should be undertaken for a better understanding.

In conclusion, impairment on respiratory function tests including pulmonary function and respiratory muscle strength tests may be a feature of mild-moderate PD and MSA patients, although without any complaint of respiratory symptoms. Most patients suffer restrictive ventilation dysfunction and decrease of respiratory muscle strength, which worsen as the disease progresses. In addition, we found a fall in diffusion capacity in MSA patients, regardless of disease severity or duration. The reduced strength of inspiratory respiratory muscles and the effect of levodopa medication may explain ventilation dysfunction. The volume of blood in capillaries supplying ventilated alveoli is more likely to contribute to the poor cardiovascular reflex performance in MSA due to gas exchanging disturbance, and the depletion of central chemosensitive glutamatergic neurons may be responsible for changes in respiratory muscle control.
